# X-ray diffraction reveals the amount of strain and homogeneity of extremely bent single nanowires

**DOI:** 10.1107/S1600576720011516

**Published:** 2020-09-23

**Authors:** Arman Davtyan, Dominik Kriegner, Václav Holý, Ali AlHassan, Ryan B. Lewis, Spencer McDermott, Lutz Geelhaar, Danial Bahrami, Taseer Anjum, Zhe Ren, Carsten Richter, Dmitri Novikov, Julian Müller, Benjamin Butz, Ullrich Pietsch

**Affiliations:** a Faculty of Science and Engineering, University of Siegen, D-57068 Siegen, Germany; b Institut für Festkörper- und Materialphysik, Technical University Dresden and Würzburg–Dresden Cluster of Excellence ct.qmat, Germany; c Department of Condensed Matter Physics, Charles University in Prague, Ke Karlovu 5, 121 16 Prague, Czech Republic; d Department of Engineering Physics, McMaster University, L8S 4L7 Hamilton, Canada; e Paul-Drude-Institut für Festkörperelektronik, Leibniz-Institut im Forschungsverbund Berlin e.V., Berlin, Germany; f Synchrotron Radiation Research, Lund University, 221 00 Lund, Sweden; g ESRF – The European Synchrotron, 71 Avenue des Martyrs, 38000 Grenoble, France; h Deutsches Elektronen-Synchrotron, PETRA III, D-22607 Hamburg, Germany

**Keywords:** nano-focused X-ray beams, nanowires, bent crystals

## Abstract

Core–shell nanowires (NWs) with asymmetric shells allow for strain engineering of NW properties because of the bending resulting from the lattice mismatch between core and shell material. The bending of NWs can be readily observed by electron microscopy. Using X-ray diffraction analysis with a micro- and nano-focused beam, the bending radii found by the microscopic investigations are confirmed and the strain in the NW core is analyzed. For that purpose, a kinematical diffraction theory for highly bent crystals is developed. The homogeneity of the bending and strain is studied along the growth axis of the NWs, and it is found that the lower parts, *i.e.* close to the substrate/wire interface, are bent less than the parts further up. Extreme bending radii down to ∼3 µm resulting in strain variation of ∼2.5% in the NW core are found.

## Abstract 2

The bending radii and strain of individual nanowires are determined using X-ray diffraction with a nano-focused beam and a dedicated diffraction theory for strongly bent crystals. Electron microscopy investigations corroborate the results.

## 1. Introduction   

Using semiconductor nanowires (NWs) one is able to design epitaxial heterostructures composed of materials with large lattice mismatch. Heterostructures can be realized along or perpendicular to the growth direction, forming axial or radial heterostructures, respectively (Larsson *et al.*, 2007 ▸; Johansson & Dick, 2011 ▸; Hilse *et al.*, 2011 ▸). For axial NW heterostructures, lattice mismatches of up to 7% – unachievable in planar growth – have been realized in the InAs/InSb system (Caroff *et al.*, 2008 ▸). For GaAs NWs, good control of growth developed over recent years includes control of their crystallographic structure (Lehmann *et al.*, 2015 ▸) and nucleation sites by using patterned substrates (Tomioka *et al.*, 2009 ▸). Control of the nucleation site is especially appealing for device applications and improves size and structure homogeneity. It also allows the investigation of properties of the same NW by various experimental techniques, as shown for example for GaAs-based core–multishell NWs by AlHassan *et al.* (2020 ▸).

The next generation of epitaxial NW heterostructures promises greater control of strain engineering. This concept was demonstrated first by growing a highly mismatched shell asymmetrically around the NW core, leading to the bending of the NW (Lewis *et al.*, 2018 ▸). In particular, GaAs core NWs were grown by molecular beam epitaxy (MBE) onto an Si(111) substrate and In_*x*_Al_(1−*x*)_As shells were preferentially grown onto one side of the core only, for example onto the (1
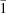
0) plane. This can be imagined as being similar to a bimetallic strip, which bends because of the different thermal expansion coefficients of two adjacent metals. In the case of NWs, in addition to different thermal properties, the lattice mismatch between core and shell material leads to bending in a predetermined direction. For the particular case described above, this means the (1
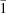
0) plane of the GaAs core is tensilely strained at the core/shell interface but compressively strained in the opposite side plane with no adjacent shell.

Using NWs, sizable strains – otherwise only obtainable by a method named strain redistribution in micro-bridges produced by complicated lithographic processing (Süess *et al.*, 2013 ▸) – can be achieved. Strain in micro-bridges, typically measured by either Raman spectroscopy (Gassenq *et al.*, 2015 ▸) or scanning X-ray diffraction (Etzelstorfer *et al.*, 2014 ▸), has been pursued to manipulate the electronic properties of materials, *e.g.* making Ge a direct band gap material (Sukhdeo *et al.*, 2014 ▸). Band gap engineering was also performed in straight GaAs/In_*x*_Ga_(1−*x*)_As core–shell NWs, and hydrostatic strains of up to 7% could be achieved (Balaghi *et al.*, 2019 ▸). Owing to the large aspect ratio of NWs, not only the absolute values of strain but also the strain gradient can be sizable in bent NWs. The change of strain across the NW diameter produces a significant impact on the electronic properties. First, the varying strain leads to a gradient of the electronic band gap and therefore a redistribution of charge carriers (Lewis *et al.*, 2018 ▸). Furthermore, it creates an additional electric field via the flexoelectric effect (Yudin *et al.*, 2014 ▸). The latter has not been observed for GaAs so far, but might be accessible in NWs with sufficiently low bending radius. Moreover, under excitation of charge carriers by a laser, this flexoelectric field becomes screened and provides a macroscopic elastic response via the converse flexoelectric effect. It is expected that the flexoelectric response may change the bending radius of the NW. Observation of the predicted effect, however, requires homogeneously bent, monophase [*i.e.* without zinc-blende/wurtzite (ZB/WZ) polytypism] NWs without plastic deformation. Here the homogeneity of the bending is related to homogeneity of shell composition as well as core and shell thickness along the entire NW length. However, during deposition of the shell material by MBE, the NW bending radius is continuously changing, and as a result the projected flux on the NW sidewall varies with time and along the axis of the NW.

In this work, we report on the use of X-ray micro-diffraction to study the bending of core–shell NWs and its homogeneity. X-ray diffraction using micro- and nano-focused X-ray beams has already been used for more than a decade to study shape and strain of nanowires either via phase retrieval (Diaz *et al.*, 2009 ▸; Robinson & Harder, 2009 ▸; Newton *et al.*, 2010 ▸) or by analysis methods supported by finite element modeling (Stankevič *et al.*, 2015 ▸; Keplinger *et al.*, 2016 ▸). Bending in NWs has been studied in InAs/InAsP and GaAs/GaInP core–shell (Keplinger *et al.*, 2010 ▸; Wallentin *et al.*, 2017 ▸) NWs, but with bending radii far larger than found in our work. Owing to the significantly stronger bending as compared to previous studies, our experiment required a modification of the diffraction setup along with a new scheme of data presentation. Moreover, current approaches of X-ray theory are limited to bending radii of the order of above 10 cm (Serrano *et al.*, 2008 ▸; Kaganer *et al.*, 2020 ▸) and no theory exists so far that is applicable to systems with such small bending radius. Using micro-focused X-ray beams, we study the bending of individual NWs and develop a suitable X-ray diffraction theory based on the kinematical approximation. The X-ray diffraction results are supported by electron microscopy investigations which image the bending. Moreover, in the diffraction analysis we directly assess not only the bending but also the strain imprinted in the NW core, which determines the electronic properties of the material.

The manuscript is organized as follows: In Section 2 we give details about the sample characteristics and experimental setup used for the X-ray diffraction measurements. In Section 3 we present our X-ray diffraction data, which are complemented by the transmission electron microscope investigations described in Section 4. Section 5 describes the X-ray diffraction theory for highly bent crystals. Finally, we discuss the results and compare the experimental data with simulations.

## 2. Experimental   

**Figure 1:**
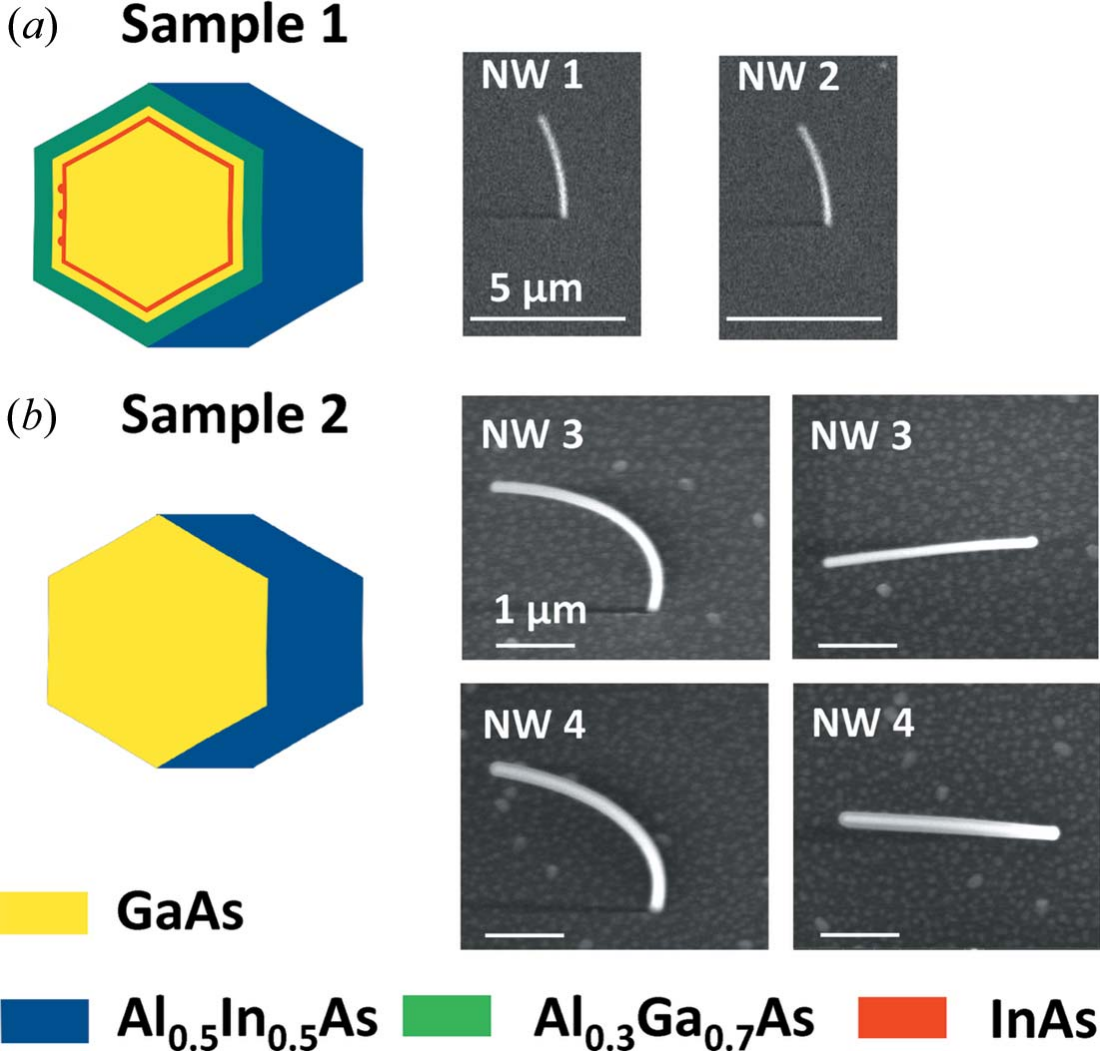
Schematic representation of the radial NW structure and scanning electron microcope images of NWs studied in this work. (*a*) Sketch and side view SEM images for sample 1 with large bending radius. (*b*) Sketch and side view (left) and top view (right) SEM images for sample 2 with smaller bending radius. Side view SEM images in (*a*) and (*b*) are recorded under a tilt angle of 30° with respect to the surface normal.

The NWs studied in this work were grown by MBE onto patterned Si(111) substrates. They consist of a GaAs core grown along the Si [111] direction, and are bent along the 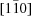 direction as a result of an asymmetric shell grown onto one side of the core only. The source fluxes were incident at an angle of 33.5° from the substrate normal. We report on two samples with bending radii, estimated from scanning and transmission electron microscopy (SEM and TEM) investigations, of approximately 8–13 µm (sample 1) and 2–3.5 µm (sample 2). Fig. 1 ▸ shows a schematic representation of the radial NW structure together with scanning electron microscopy images of the particular NWs investigated by X-ray diffraction. The NWs of sample 1 have considerably larger bending radius in comparison with NWs from sample 2, as can be seen in the SEM images in Fig. 1 ▸. Sample 1 [whose growth was reported earlier by Lewis *et al.* (2018 ▸)] is composed of GaAs/InAs/GaAs/Al_0.3_Ga_0.7_As/Al_0.5_In_0.5_As core–multishell NWs with a 75 nm GaAs core, a 2–3 nm InAs shell including quantum dots (QDs), a 5 nm GaAs shell, a 10 nm Al_0.3_Ga_0.7_As shell and an outermost partially grown shell of Al_0.5_In_0.5_As with a nominal thickness of 40 nm. Note that the thickness of the core denotes the separation between opposing facets, while the shell thicknesses correspond to the thickness of the shell layer on a given facet. The complicated radial structure is beneficial for the optical properties of the NWs that were studied by Lewis *et al.* (2018 ▸). Owing to the small thickness of the InAs shell and QDs, we expect that they can be neglected for the present study. Sample 2 consists of a nominally 40 nm thick Al_0.5_In_0.5_As partial shell grown onto a GaAs core with 7 nm diameter. Because of the different ratio of effective core versus asymmetric shell thickness, the NWs of sample 2 are more strongly bent, *i.e.* have a smaller bending radius.

The bending radius of the NWs can be extracted from SEM images by overlaying the NW axis with ellipses and adjusting the radius to fit the observations. Because the tilt angle with respect to the surface normal is 30°, ellipses with an aspect ratio of 2:1 have to be used. For NW1 and NW2 of sample 1 we obtain bending radii of 10–13 µm. In contrast, NW3 and NW4 have considerably greater bending and correspondingly smaller radii of 2.5–3.5 µm. The process of overlaying ellipses on the SEM images, in particular for the NWs of sample 2, does not allow us to obtain a perfect match for the full wire using only one bending radius. The values determined using this method therefore represent the average bending of the full wire. To obtain a more local bending radius from the SEM images we determined the position of the NW’s center line along the NW and numerically calculated the local bending radius using finite difference differentiation. Using this method we obtain radii consistent with those mentioned above for the central parts of the NWs. However, especially for the bottom parts, the bending radii exceed the given ranges, indicating that the bending closer to the substrate interface is significantly lower.

**Figure 2:**
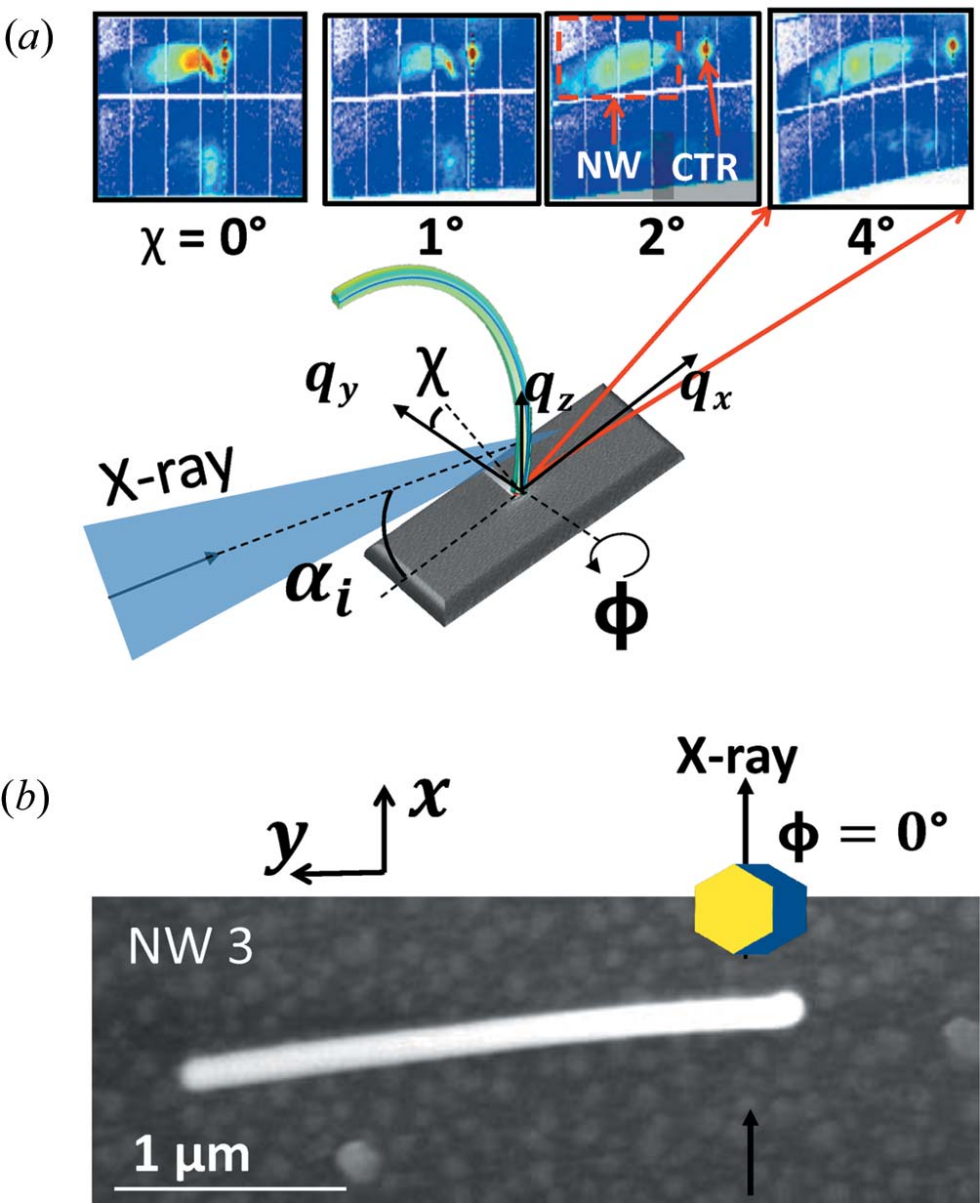
(*a*) Sketch of the experimental setup. The focused X-ray beam illuminates part of the NW and produces a diffraction signal, as illustrated by the examples of detector images shown as insets. Typical detector images include diffraction signal of the illuminated NW and the substrate’s crystal truncation rod (CTR). (*b*) Diffraction geometry with respect to the NW cross section at the sample azimuth ϕ = 0° in comparison with a top view SEM image.

While from SEM images one can determine only the bending of the NW shape, X-ray diffraction allows one to study the effect on the atomic distances within the NW. Our diffraction studies were performed using micro-focused X-ray beams in order to obtain the signal of (parts of) individual NWs. In particular, NW1 and NW2 were measured with a coherent X-ray beam at beamline ID01 of ESRF (Grenoble, France), focused down to an FWHM of 0.23 × 0.3 µm [vertical (V) × horizontal (H)] and with a photon energy of 9 keV. NW3 and NW4 were measured at beamline P23 of PETRA III, DESY (Hamburg, Germany), with an X-ray beam focused down to an FWHM of 0.8 × 3 µm (V × H) and photon energy of 10 keV. As the two experimental setups are qualitatively similar, we present the general experimental setup in Fig. 2 ▸. A convergent X-ray beam is positioned at various points along the NW and the corresponding diffraction data are collected. Examples of detector images are shown in Fig. 2 ▸(*a*). The images typically include broad signals originating from the NW and a sharp crystal truncation rod from the substrate. In order to present the data of the bent NWs over the entire length, we choose a reciprocal-space coordinate system aligned with the single-crystalline substrate. The *q* space is defined such that the *q*_*z*_ vector is along the substrate’s [111] direction (surface normal). The *q*_*x*_ direction coincides with the X-ray beam direction at zero goniometer angles and corresponds to the [11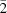] direction of the substrate. Therefore, *q*_*y*_ is along the [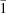
10] direction of the substrate and is also roughly within the plane in which the NWs bend.

Our studies focus on diffraction from the GaAs {111} lattice planes, or the equivalent {0002} WZ phase lattice planes, which for the bottom part of the NWs are parallel to the {111} planes in the substrate. The diffraction signal therefore is located along the *q*_*z*_ direction with vanishing *x*, *y* components. Given the photon energies of 9 keV (NW1 and NW2) and 10 keV (NW3 and NW4) the Bragg condition for the GaAs volume near the substrate is therefore fulfilled at angles α_i_ = 12.18 and 10.95°, respectively. Using these incidence angles and corresponding detector angles, we located the bottom parts of the NWs by scanning the sample surface through the beam at the Bragg condition via the *x*, *y* translation stages.

The bending of the upper parts of the NWs causes the diffraction signal of the corresponding parts to tilt. Within our chosen reciprocal-space coordinate system, this tilt is predominantly along the *q*_*y*_ direction. A small *q*_*x*_ component exists only because of a slight offset of the plane in which the NWs bend [see for example Fig. 2 ▸(*b*)]. So in order to collect diffraction signal from these bent parts, in addition to the beam location on the sample, the goniometer angles need to be adjusted. Several possibilities exist to adjust the goniometer angles. Given the experimental possibilities at beamlines ID01 and P23 we had to choose two different strategies: At ID01 (NW1 and NW2) we used the sample azimuth ϕ [see Fig. 2 ▸(*a*)] and the corresponding detector rotation to follow the diffraction signal along the NWs. On the other hand at beamline P23 (NW3 and NW4) we used the sample tilt χ to maintain the Bragg condition for the investigated segment without any change of the detector position. For NW3 the diffraction signal was recorded for tilt angles from 0 up to 50° with a step size of 0.5–3°, always ensuring that some overlap of subsequent reciprocal-space maps (RSMs) existed. Insets in Fig. 2 ▸(*a*) show average detector images of rocking curve measurements for various tilt angles. Because NW3 was grown slightly tilted with respect to the *y* axis in addition to χ, a small correction of the sample azimuth (ϕ) had to be used to align NW3 into the diffraction condition. The SEM image in Fig. 2 ▸(*b*) corresponds to the case of ϕ = 0°, which shows that for different parts of the NW different ϕ angles have to be used. In both experiments the X-ray beam illuminates the NW roughly from the direction perpendicular to the small facets of the NWs having irregular octagonal shape resulting from the asymmetrically grown shell [see inset in Fig. 2 ▸(*b*)]. While this condition is fulfilled for the full NW using the geometry at P23, it is only true when studying the bottom parts at beamline ID01. Two-dimensional detectors at distances of 569 mm (4 chip MaxiPix detector) and 1020 mm (2D Lambda detector) were used at ID01 and P23, respectively. Three-dimensional RSMs were recorded either by scanning the incidence angle (P23) or at fixed incidence angle but varying the X-ray energy between 8.5 and 9.5 keV with a step size of 0.04 keV (ID01). At ID01, preliminary data processing was performed using the *XSOCS* package (Chahine *et al.*, 2014 ▸).

**Figure 3:**
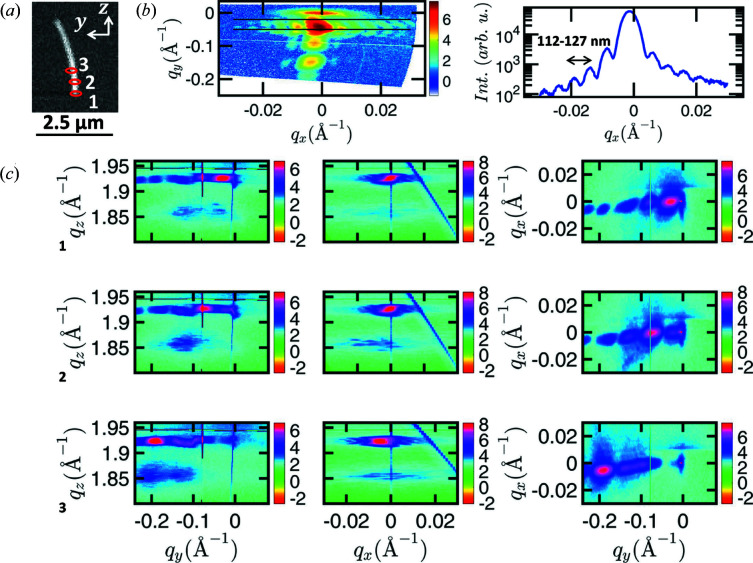
(*a*) SEM image of NW1 with the central X-ray beam position of measurements 1, 2 and 3 marked. (*b*) *q*_(*x*,*y*)_ plane extracted from the 3D RSM at the GaAs 111 Bragg reflection recorded at position 1. The signal between the black lines is shown in the line plot on the right and exhibits thickness oscillations corresponding to a size of 120 ± 8 nm. (*c*) 2D projections of 3D RSMs measured at the three different positions along the growth axis of NW1. For each position the *q*_(*z*,*y*)_, *q*_(*z*,*x*)_ and *q*_(*x*,*y*)_ projections are shown by filled contour plots with a logarithmic intensity scale.

Prior to NW measurements at beamline ID01 of the ESRF, the X-ray wavefront was characterized by means of 2D ptychography in the forward direction using a Siemens star test sample. The X-ray wavefront was reconstructed using the *PyNX* software (Mandula *et al.*, 2016 ▸) and is presented in supplementary Fig. S1. It can be seen that, besides the central main peak, the X-ray intensity displays tails expanding in real space to around 4 µm along the vertical directions: the main maximum in the center of the beam and four to five side maxima. Interaction of these maxima with the highly bent crystal structures will be discussed later during the explanation of the RSMs presented in Fig. 3.

## 3. Micro-focus X-ray diffraction data   

In this section the recorded RSMs from NW1 and NW2 are presented and discussed; the individual RSMs from NW3 and NW4 are presented in the supplementary material.

Fig. 3 ▸ shows the strategy of mapping the NW’s GaAs 111 Bragg reflection at beamline ID01 for sample 1. The NW is scanned by the X-ray beam at different positions along the growth axis [see Fig. 3 ▸(*a*)]. At each position a 3D RSM is recorded. In the case of position 1, the *q*_(*x*,*y*)_ slice taken from the 3D RSM at the main maximum shows thickness fringes along the *q*_*x*_ direction corresponding to a size of around 112–127 nm [see Fig. 3 ▸(*b*) and Fig. 1 ▸(*a*)]. This is in good agreement with the NW dimensions given in Section 2, from which a nominal distance between the upper and lower blue facets [see Fig. 1 ▸(*a*)] of ∼126 nm is expected.

Examples of the projections of the 3D RSM onto the *q*_(*z*,*y*)_, *q*_(*z*,*x*)_ and *q*_(*x*,*y*)_ planes are shown in Fig. 3 ▸(*c*) for the different positions along the NW. As seen at the bottom part of the NW (position 1), the projection of the RSM onto the *q*_(*z*,*y*)_ plane has a maximum at *q*_*y*_ = 0 Å^−1^. Here we observe an envelope function with clear maxima and minima due to the wavefront of the X-ray beam illuminating the NW along the vertical direction. The interaction of the (111) planes, with varying tilt in the NW, with the coherent focused X-ray beam leads to complex scattering and interference patterns originating from different locations on the NW. The resulting scattering pattern in Fig. 3 ▸(*c*1) for *q*_(*z*,*y*)_ and *q*_(*x*,*y*)_ projections can be explained in the following way: (1) The main maximum of the X-ray beam’s wavefront is aligned with the bottom part of the NW, where the 111 planes are parallel to the substrate surface. From this the peak near *q*_*y*_ = 0 Å^−1^ results. (2) Parts of the NW further away from the substrate surface are illuminated by the side maxima (tails) of the X-ray beam, which are shown in Fig. S1. Since the segments of the wire illuminated by the tails are tilted, side fringes of the illumination function cause minima at *q*_*y*_ ≃ −0.08 Å^−1^ and *q*_*y*_ ≃ −0.17 Å^−1^ as well as maxima at around *q*_*y*_ ≃ −0.1 Å^−1^ and *q*_*y*_ ≃ −0.2 Å^−1^. These maxima originate roughly from positions 2 and 3 on the NW. This interpretation is corroborated by the patterns shown in Figs. 3 ▸(*c*2) and 3 ▸(*c*3), which are recorded at positions 2 and 3 and have their corresponding main maximum near the side maxima observed in Fig. 3 ▸(*c*1). On the basis of the arguments above, the reconstructed wavefront of the X-ray beam can be used to retrieve the illumination position of the X-ray beam on the NW. For this purpose we use the known distance between the maxima of the wavefront in real space. Considering the experimental geometry, we recalculate this spacing as a distance along the NW growth axis. Accordingly, two neighboring maxima in the diffraction pattern originate from segments located around 250 nm apart from each other along the NW growth axis. Note that the distance determined in this way is significantly less affected by time drifts as compared to RSMs recorded for different motor positions since both the different *q*-space position and the real-space position are obtained from the same measurement.

The bending radius of the NW crystal was calculated from the distance between two NW segments and their tilt angle, determined from the center of mass of maxima in the envelope function. In this way we obtain a bending radius of ∼8–12 µm. The large spread in values originates from the fact that the spacing of the fringes is not equal in Fig. 3 ▸(*c*). Note that an anomaly near *q*_*y*_ = −0.1 Å^−1^ consistently appears in the particular data shown in Fig. 3 ▸(*c*). This is probably the result of some major defect, which will locally also change the bending radius. While the radius determined by X-ray diffraction determines the local bending of the crystal, where the measurement was performed, the radius determined by SEM corresponds to an average bending radius of the NW shape. Nevertheless, a good agreement between these two values is found.

In the *q*_(*z*,*y*)_ plane shown Fig. 3 ▸(*c*), the diffraction signal seen around *q*_*z*_ ≃ 1.85 Å^−1^ corresponds to non-pseudomorphic defective shell material grown on the wire. This can be concluded from the evolution of the signal for different illumination positions seen in Fig. 3 ▸(*c*) in the *q*_(*z*,*y*)_ and *q*_(*z*,*x*)_ projections. The width of the NW peak along the radial *q* direction is large and lies between the known peak positions of wurtzite and zinc-blende crystalline structures, which can be present in the NW at the same time (Jacobsson *et al.*, 2015 ▸). This hinders our study of the NW in terms of crystalline phase distribution along the NW growth axis from these data.

**Figure 4:**
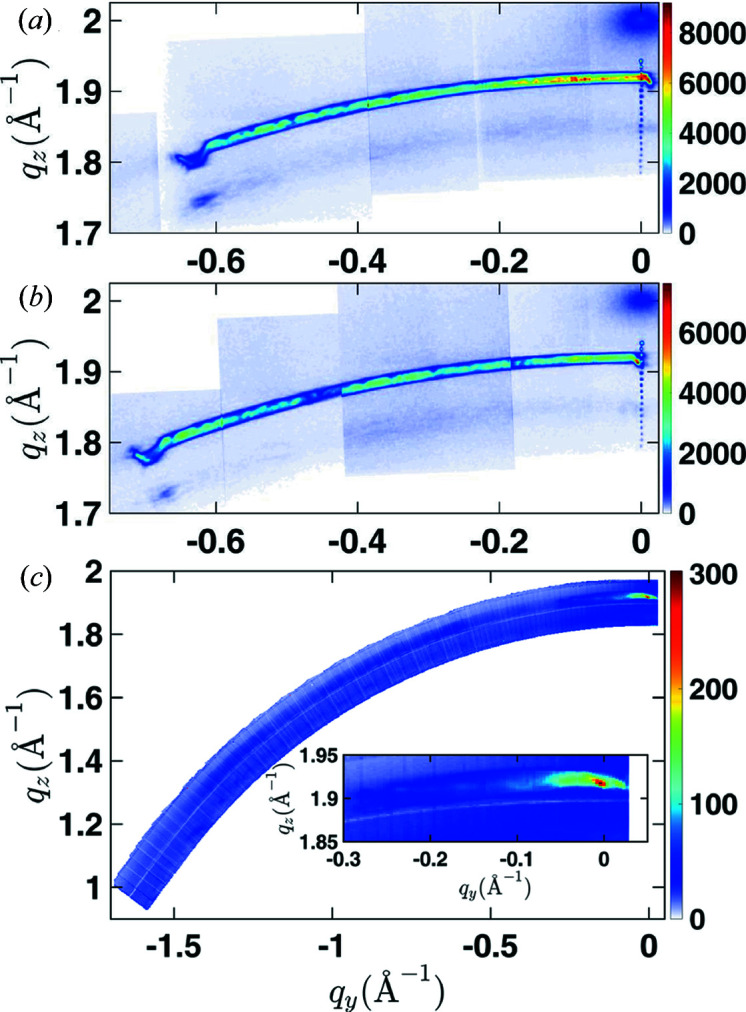
The projections of the combined RSMs for (*a*) NW1 and (*b*) NW2 of sample 1 and (*c*) NW3 of sample 2. The signal from the bent NWs spreads out along a segment of the Debye ring. An inset in panel (*c*) shows a magnification of the signal near *q*_*y*_ = 0 Å^−1^.

Owing to the small beam size and strong bending, only part of the NW contributes in a single measurement. An RSM for the entire NW is obtained only by combination of several measurements like those shown in Fig. 3 ▸. For this purpose, RSMs from many different real-space positions as well as for different angular positions have to be combined. In the case of NW1 and NW2, more than 36 000 individual 3D RSMs were analyzed and combined together. Projections of the resulting RSMs for NW1 and NW2 are shown in Figs. 4 ▸(*a*) and 4 ▸(*b*). Combining data recorded at different locations washes out the coherent diffraction patterns observed in Fig. 3 ▸ because all the segments of the NW fulfill the Bragg condition individually during the RSM scanning. The result is the observation of diffraction signal distributed along a segment of a Debye ring, which will be discussed in more detail in Section 6. An anomaly in the signal near its termination in Figs. 4 ▸(*a*) and 4 ▸(*b*) originates from diffraction of the very top part of the NW.

For NW3 and NW4 of sample 2, projections of individual RSM measurements are presented in Fig. S2 in the supplementary material. In contrast to the data shown in Fig. 3 ▸, the patterns show no diffraction speckles. This difference is likely to be caused by a combination of multiple effects: First, the focal spot at beamline P23 used for these measurements was significantly larger and therefore the central maximum of the focal spot illuminates a considerable fraction of the NW. Second, NW3 and NW4 have much lower bending radii, which suggests that only a short segment of the NW can fulfill the Bragg condition within one reciprocal-space map. Third, while the beam at ID01 is highly coherent this is not the case for the beam used at P23. Forty-two RSMs of NW3 recorded for different sample tilts χ were collected and combined to create the RSM shown in Fig. 4 ▸(*c*). At *q*_*y*_ = 0 Å^−1^ the Bragg peak of the GaAs 111 reflection is relatively sharp and intense. However, its intensity decreases while its FWHM along the radial *q* coordinate increases up to *q*_*y*_ = −0.09 Å^−1^. The strong intensity near *q*_*y*_ = 0 Å^−1^ originates from the bottom part of the NW, which is less bent compared with parts further up. This lower bending results in a higher material volume which simultaneously satisfies the Bragg condition and therefore causes the strong signal.

In the range of −1.5 < *q*_*y*_ < −0.09 Å^−1^ the intensity variations are probably the result of slight misalignment of the beam position away from the NW. Owing to time limitations it was not possible to collect diffraction data from the full NW. Although hardly visible in Fig. 4 ▸(*c*), the signal extends beyond the measurement range. From the bending observed in the SEM images one would expect the signal to continue along the Debye circle until *q*_*z*_ ≃ 0 Å^−1^.

In the measurements shown in Fig. 4 ▸, the FWHM of the Bragg peak along the radial *q* coordinate is related to the different lattice plane spacings inside the GaAs NW core, *i.e.* the strain variation in the NW. As we show later, it is therefore inversely proportional to the bending radius. The widening of the diffraction signal at lower *q*_*y*_ values in Fig. 4 ▸(*c*) could be related to a variation of the local bending radius. This will be discussed in more detail after we introduce a theoretical approach which allows us to quantify the strain gradient/bending.

## 4. Transmission electron microscopy   

In order to support the X-ray diffraction results, we performed high-resolution TEM investigations in cross-section geometry. For this purpose, a few NWs of samples 1 and 2 were scratched carefully from the silicon substrate and were deposited onto a lacy carbon support grid. The TEM analysis was performed by using an FEI Talos F200X operated with an acceleration voltage of 200 kV on selected NWs lying nearly flat on the support film, *i.e.* the bending plane was oriented perpendicular to the viewing direction.

**Figure 5:**
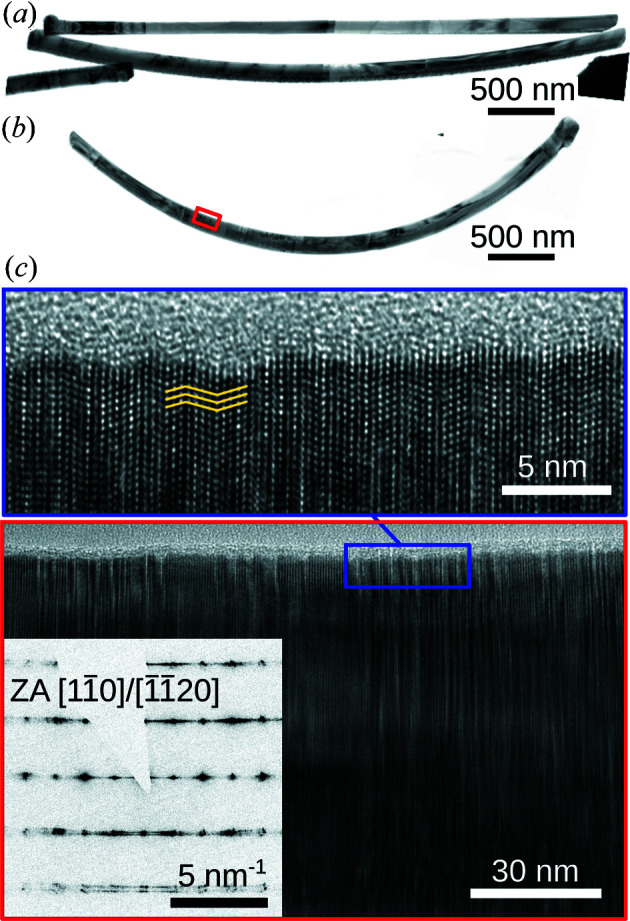
Transmission electron micrographs of NWs from sample 1 (*a*) and sample 2 (*b*). From nanowires that have their bending plane parallel to the imaging plane, the bending difference between the samples [*cf*. (*a*) and (*b*)] is evident. A high-resolution image of the region marked by the red rectangle in (*b*) is shown in (*c*). By further zooming in to the region marked by the blue rectangle, planar defects can be seen. In (*c*), the yellow lines highlight a twinned region. The inset in (*c*) shows an electron diffraction pattern recorded along the 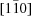 cubic or equivalent 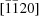 hexagonal zone axis, respectively.

Fig. 5 ▸ shows examples of low- and high-resolution images of samples 1 and 2. In Fig. 5 ▸(*a*), stitched images of two complete NWs of sample 1 are shown. The upper NW has its bending plane parallel to the viewing direction and was therefore disregarded in the analysis. In contrast, the lower NW is lying flat on the support grid such that a bending radius of 8–9 µm can be measured. For the NW of sample 2 shown in Fig. 5 ▸(*b*), we identify a change of the bending radius from ∼3 µm on the left to ∼2.3 µm on the right of the image. Note that the right-hand side corresponds to the top of the NW. Despite the fact that the NWs have been randomly scratched from the Si substrate, the bending radius found here agrees reasonably well with the radii that were found in the X-ray analysis.

In addition to the bending, it is possible to identify local crystallographic and defect structure information on the NWs. For sample 1, all inspected NWs appear very homogeneous in the middle and bottom parts of the NW, with a very low density of planar defects. The region close to the top, just below the crystallized droplet, shows a sequence of fast changing ZB and WZ phase units. In contrast to this, NWs of sample 2 [Figs. 5 ▸(*b*) and 5 ▸(*c*)] are highly defective in the lower half, while the upper part is almost defect free. This highly defective region has been found in nearly all inspected NWs but with different extent and position along the NW. The high number of defects in the defective part becomes obvious by the streaking of diffraction spots seen in the inset of Fig. 5 ▸(*c*) and in Fig. S3 in the supporting information. The images show that a large number of planar defects and phase changes are present in the sample. The NW shown in Fig. 5 ▸(*b*) is mainly composed of the WZ phase. Other NWs of the same sample probed by TEM also show the ZB phase with a similar volume fraction of the defective region. About 20% of highly bent NWs did not show this defective region.

Fig. S3 shows that, in agreement with the expectation from NW growth, the local [111] or [0001] crystal direction is always aligned with the NW axis. Since the determination of the bending radius from X-ray diffraction measurements presented above relies on the crystal orientation, it is important to obtain an independent proof of this aspect.

## 5. Diffraction theory of bent NWs   

In this section we simulate diffraction RSMs of a bent NW. The aim of this simulation is to qualitatively demonstrate the influence of bending on the shape of the diffraction maximum. In addition to the kinematical approximation we make the following assumptions:

(1) The far-field limit applies. The validity of this assumption is proven by the calculation of the phase factor of the Fresnel propagator 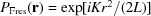, where *K* = 2π/λ is the wavevector length and *L* is the sample–detector distance. In our experimental arrangement, the exponential term of this propagator is smaller than 10^−4^.

(2) The NW is ideally circularly bent and its circular axis lies in the *yz* plane perpendicular to the sample surface. This assumption makes the simulation much easier: the differences between the actual and circular NW shapes could affect the tails of the diffraction maximum. The incident X-ray beam lies in the *xz* plane and we calculate the reciprocal-space distribution of the diffracted radiation (reciprocal-space map) in plane *q*_*y*_ *q*_*z*_ parallel to *yz*.

(3) The NW cross section is circular. Possible facets on the NW sidewalls would create streaks, which, however, are not visible in the *q*_*y*_ *q*_*z*_ reciprocal plane.

(4) The elementary unit cells of the NW structure are not deformed by bending, *i.e.* the structure factors of individual reflections are not affected by bending either. A modification of the structure factor by bending leads to a change in the diffracted intensity; however, the shape of the diffraction maximum in reciprocal space is not affected by this simplification.

We denote by *R* the bending radius and 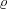 the radius of the wire cross section. The position vector of an elementary unit cell is
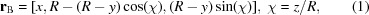
where 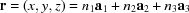 is the position vector of the same cell in a non-bent NW, *a*_1,2,3_ are the basis vectors and *n*_1,2,3_ are integers. Furthermore, we denote by 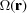 the shape function of the non-deformed NW (unity inside the NW volume and zero outside of it).

Under the assumption above, the wave scattered into the point 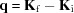 of reciprocal space is (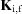 are the wavevectors of the primary and scattered beams)
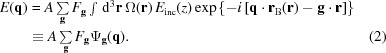
Here *A* is an uninteresting factor very slightly dependent on *q*, 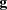 are the vectors of the lattice reciprocal to the non-deformed NW lattice, 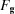 is the structure factor of reflection 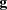, and *E*_inc_(*z*) is the amplitude of the incident wave. We assume that this amplitude depends only on the vertical coordinate *z* and the cross-section profile of the incident beam is Gaussian:
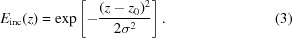
The FWHM of the incoming beam along the *z* axis is proportional to the parameter σ: 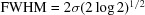.

The integrals in the amplitudes 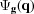 can be partially evaluated and we obtain
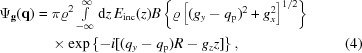
where
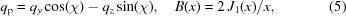
with *J*_1_(*x*) the Bessel function of first order. The remaining integral over *z* has to be evaluated numerically. In order to avoid numerical complications at the NW ends we assume that the NW is much longer than the irradiated footprint determined by *E*_inc_(*z*). Therefore the integration limits can be expanded to  ±∞.

For a rough estimation of the diffraction maximum position in the *q*_*y*_ *q*_*z*_ plane we can approximate the integral in equation (2) by the stationary phase method; in this approach we ignore the *x* integration and calculate the integral only in the *y* *z* plane. The stationary points of the phase
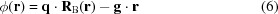
are
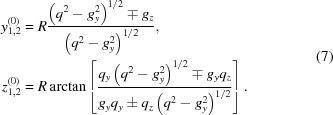
Furthermore, we define the Hessian of the phase function 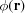 and calculate its determinant in the stationary points. Both points yield the same value:
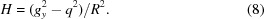
The stationary phase approximation of the integral 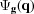 is then
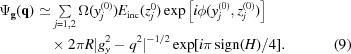
This formula allows us to estimate the position 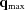 of the diffraction maximum in the *q*_*y*_ *q*_*z*_ plane. The maximum occurs at the point at which 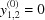 and 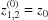. This condition yields an obvious result, namely the angle between the vectors 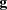 and 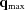 equals χ.

The maxima of the integrals 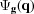 for different 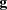 almost do not overlap, so that we can neglect the sum 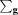 in equation (2), writing
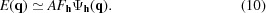
Here 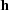 denotes the reciprocal-lattice vector lying closest to 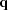.

**Figure 6:**
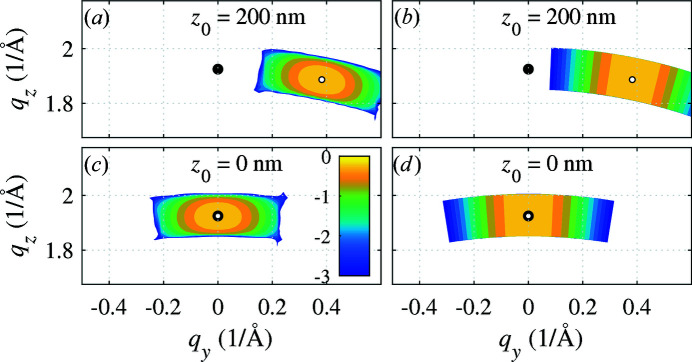
The reciprocal-space maps calculated for symmetrical diffraction 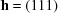 using the exact kinematical formula (2) [panels (*a*) and (*c*)] and the stationary-phase method in equation (7) [(*b*) and (*d*)] for various positions *z*_0_ of the primary beam (parameters of the graphs). The filled and empty circles denote the end points of the non-rotated reciprocal-lattice vector 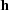 and the rotated vector 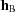, respectively. The intensity is displayed logarithmically. Color bar ticks are labeled with the decadic exponents of the intensity relative to the intensity maximum.

**Figure 7:**
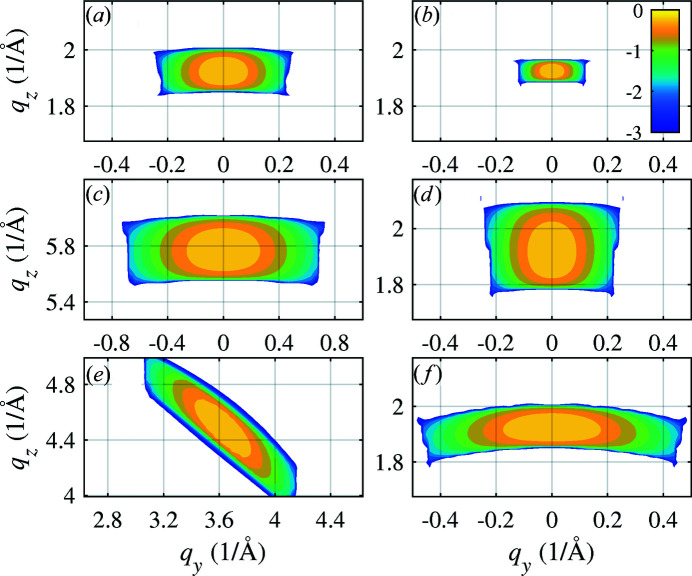
The reciprocal-space maps calculated using equation (2) for 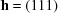 [panels (*a*), (*b*), (*d*) and (*f*)], 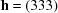 (*c*) and 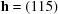 (*e*). In (*b*) the simulation was carried out for two times larger bending radius, panel (*d*) shows the map calculated for two times larger NW radius, and in (*f*) the map shows the data for two times larger FWHM of the primary beam. The intensity is displayed logarithmically. Color bar ticks are labeled with the decadic exponents of the intensity relative to the intensity maximum.

In Figs. 6 ▸, 7 ▸ and 8 ▸ we present examples of the results of numerical simulations. In Fig. 6 ▸ and in panels (*a*), (*c*) and (*e*) of Fig. 7 ▸ we consider an NW with a radius of 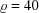
 nm and a bending radius *R* = 1 µm. The width parameter of the incident beam was chosen to be σ = 60 nm so that the FWHM of the beam was 100 nm. Panels (*b*), (*d*) and (*f*) of Fig. 7 ▸ show the calculated maps for *R* = 2 µm, 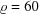
 nm and FWHM = 200 nm. In Fig. 6 ▸, the end points of 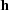 and 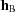 are displayed as filled and empty circles; the diffraction maximum indeed lies at 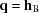. The figure demonstrates that for a qualitative estimate of the maximum position and shape the simple stationary phase calculation is fully sufficient. The diffraction maxima are arc shaped, elongated in the direction perpendicular to the diffraction vector 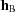, and rotate by an angle χ with respect to 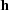 as a result of bending. The arc length and width are inversely proportional to the bending radius *R*_B_ [compare the panels (*a*) and (*b*) in Fig. 7 ▸]; the arc length is proportional to the FWHM of the primary beam, *i.e.* to the length of the irradiated NW segment [panels (*a*) and (*f*)].

**Figure 8:**
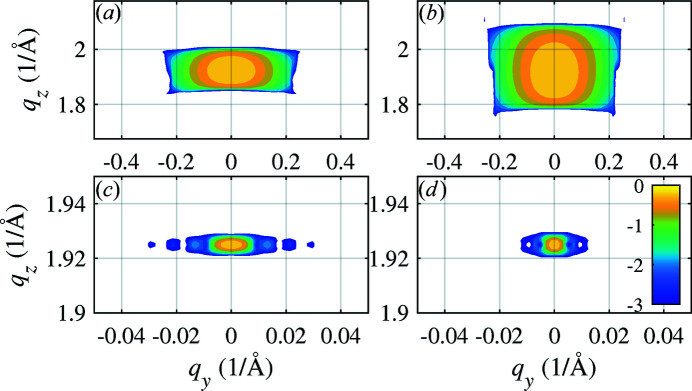
The (111) reciprocal-space maps calculated for small and large NW radii and strong bending (*R* = 1 µm) in (*a*) and (*b*), and for the same NW radii and slight bending (*R* = 100 µm) in (*c*) and (*d*). The intensity is displayed logarithmically. Color bar ticks are labeled with the decadic exponents of the intensity relative to the intensity maximum.

Interestingly, the width of the arc is proportional to the NW radius 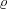 [Figs. 7 ▸(*a*) and 7 ▸(*d*)]. This counter-intuitive behavior demonstrates that the size of the diffraction maximum is determined mainly by strain and not by the size of the irradiated NW volume. This effect is demonstrated in Fig. 8 ▸, where we compare reciprocal-space maps calculated for 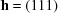 for two NW radii (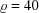 and 80 nm) and strong bending *R* = 1 µm [panels (*a*) and (*b*)] and slight bending *R* = 100 µm [panels (*c*) and (*d*)]. While in the case of strong bending the arc width is proportional to 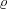, in the case of slight bending the arc length is inversely proportional to 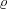, and the arc width is inversely proportional to the beam FWHM.

**Figure 9:**
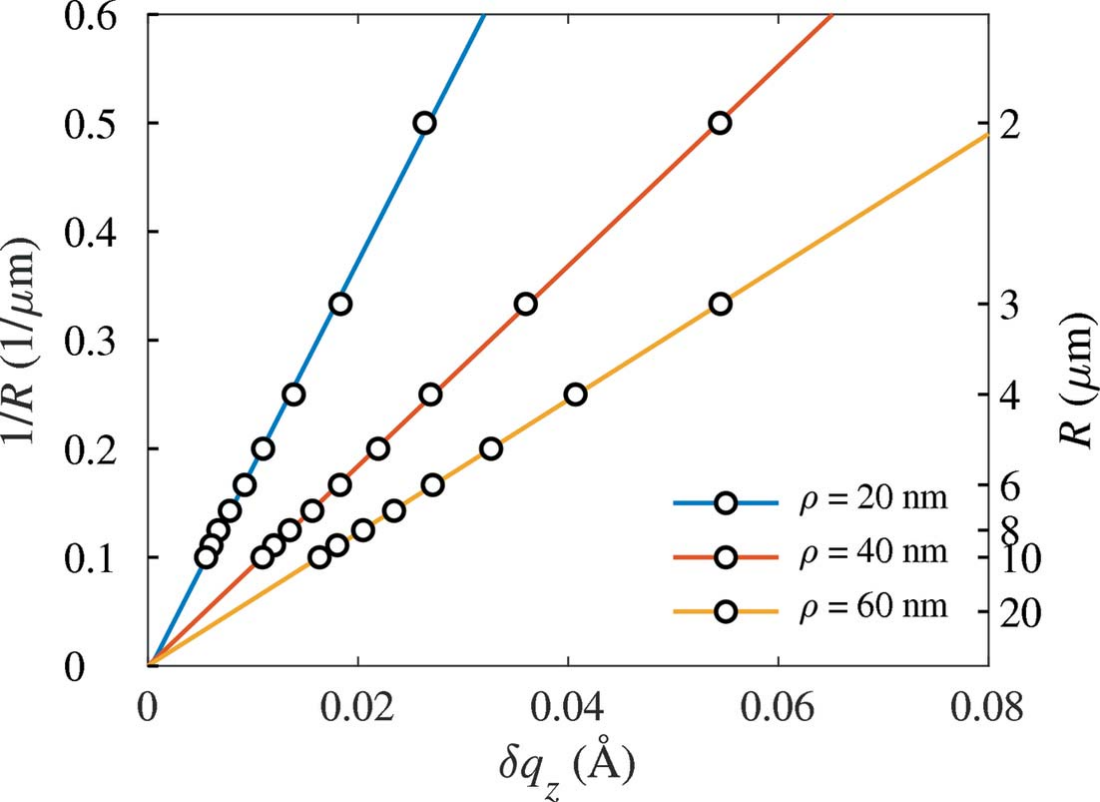
The linear dependence of the reciprocal bending radius on the width of the 111 diffraction maximum in the *q*_*z*_ direction calculated for various NW radii (parameters of the curves). The circles represent the data obtained by calculation using equation (2); the lines are their linear fits.

The arc width δ*q*_*z*_ can be used for an easy determination of the bending radius *R*. This is demonstrated in Fig. 9 ▸, where we have plotted the inverse bending radius 1/*R* as a function of δ*q*_*z*_ determined by numerical calculation of the 111 Bragg spot using equation (2) (points). The dependence is almost linear; the straight lines in the figure show the linear approximation of the 1/*R* versus δ*q*_*z*_ dependence. The slope of this dependence decreases with increasing NW radius 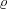.

## 6. Results and discussion   

Using the measured data presented above as well as the theory introduced in the previous section, we will further process the experimental data and compare them with simulations to assess the strain state of the nanowires.

**Figure 10:**
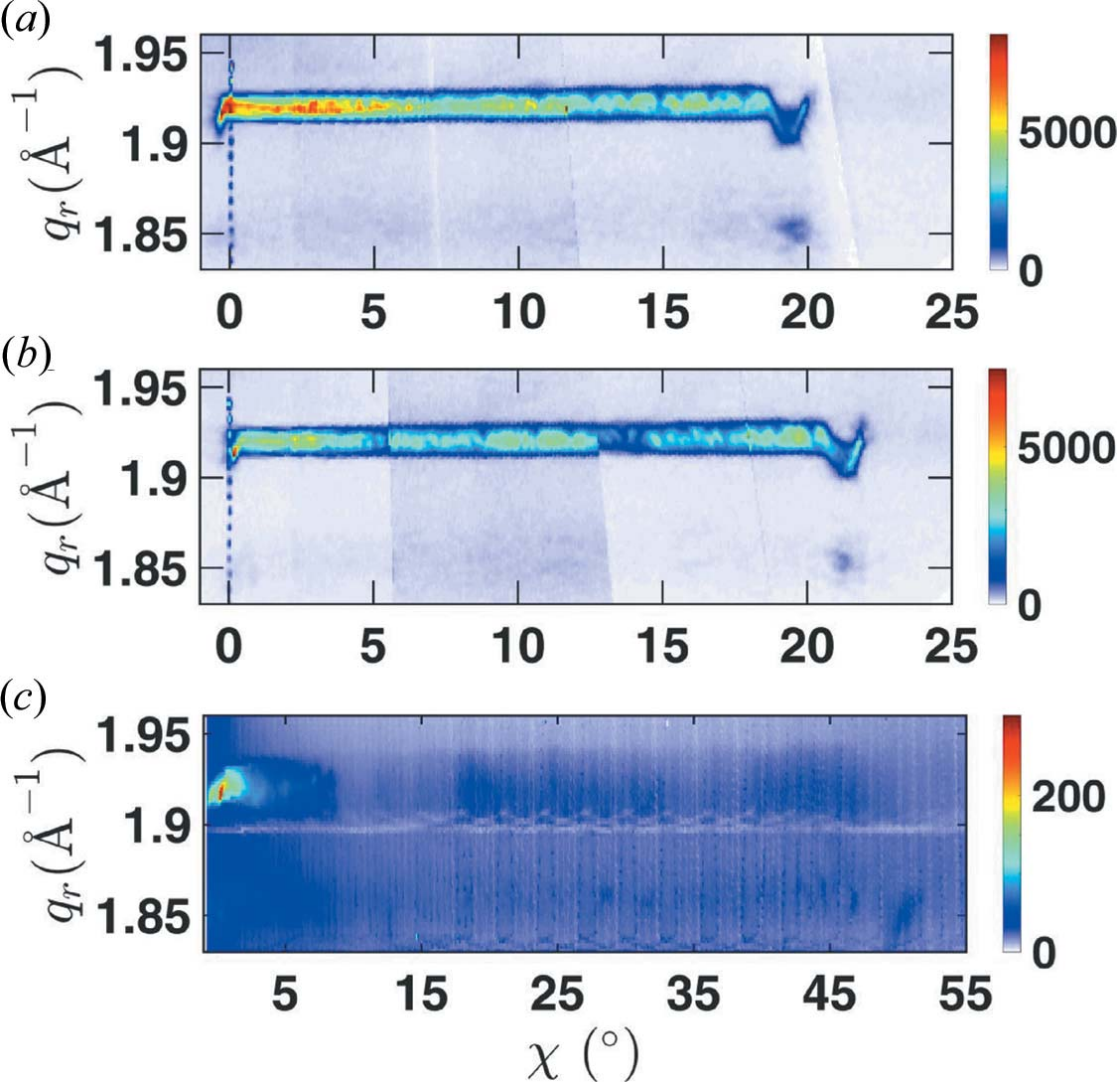
Radial integration of the combined RSMs of (*a*) NW1 and (*b*) NW2 of sample 1 and (*c*) NW3 of sample 2. Data are plotted versus the angle χ which specifies the tilt with respect to the substrate surface.

For this purpose, we replot the experimental data from Fig. 4 ▸ using the radial coordinate 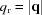 and the tilt angle χ as coordinates in Fig. 10 ▸. It can be seen that in the case of NW1 and NW2 of sample 1 with higher bending radius the signal extends up to χ ≃ 20 and 22°, respectively. Around χ = 18° for NW1 and χ = 21° for NW2 a sudden change is detected in the RSM, which we associate with the top segment of the NW. This segment is likely to have a different chemical composition, since it might originate from axial wire growth during the shell growth, similarly found by AlHassan *et al.* (2018 ▸). In Fig. 10 ▸(*c*) showing data of NW3 of sample 2 no such anomaly from the top of the wire is observed, since the top part according to the SEM images is tilted almost 90° far beyond the end of the measurement range. As concluded from the simulations, the different tilt range of the signal in Figs. 10 ▸(*a*) and 10 ▸(*b*) could be a result of differences in either the bending radii or the lengths of the NWs. Since the lengths determined from the SEM images shown in Fig. 1 ▸(*a*) are rather similar, the likely explanation is that the bending radii of these wires are slightly different. A close inspection of the SEM images in agreement with the higher tilt range of NW2 seen in Fig. 10 ▸(*b*) suggests that the top of NW2 is more bent as compared with NW1.

**Figure 11:**
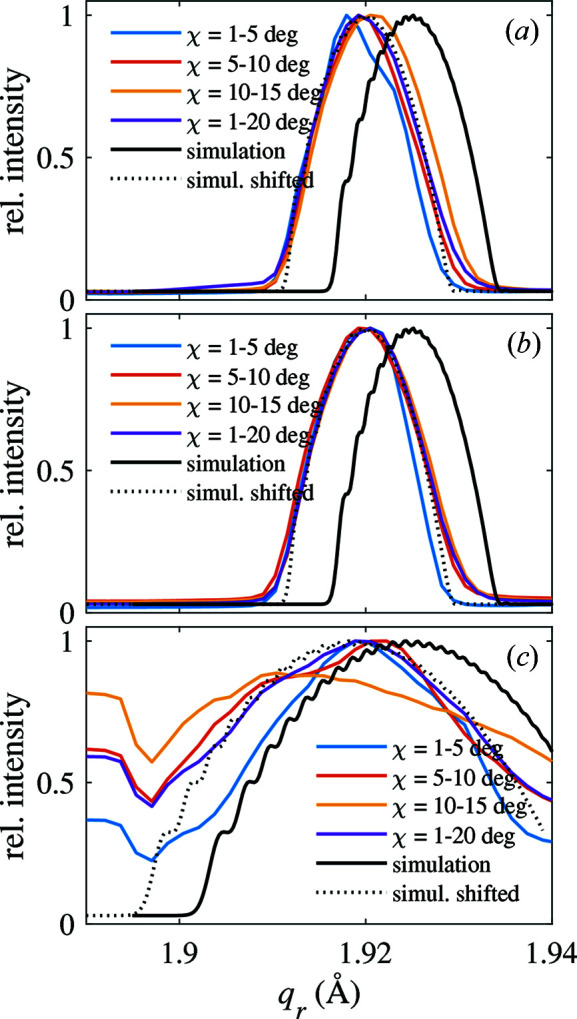
Comparison of experimentally measured intensity profiles (color lines) along the *q*_r_ direction with respect to calculation based on bent circular NWs (full and dotted black lines). Panels (*a*), (*b*) and (*c*) show data for NW1 and NW2 of sample 1 and NW3 of sample 2, respectively. Various tilt integration ranges indicated in the figure legend were used to obtain these curves from data shown in Fig. 10 ▸.

To further compare the diffraction signal of the NWs, we obtain line cuts along the radial direction averaged over different ranges of tilt χ and compare them in Fig. 11 ▸. In agreement with the expectations from our model calculations, the higher bending radius of NW1 and NW2 of sample 1 causes their signal to be considerably narrower than that of NW3 of sample 2. Another observation is that the width of the curves gets slightly wider when it is extracted from higher tilt values. This means that the bending radius is not entirely homogeneous along the NW axis. Considering that the base of the NW is fixed epitaxially to a rigid support, it makes sense that the bending at the bottom needs to develop and can reach its highest values only a certain distance away from the wire–substrate interface. Since the deposition geometry also gets highly complicated and evolves during growth, one also expects an inhomogeneity in the shell thickness along the NW growth axis. Both effects support a change of the bending for different positions along the NW.

In order to understand the contribution of the individual effects, we performed simplified model calculations of the shell growth process as described by Lewis *et al.* (2018 ▸). Since the deposition rate on the NW sidewall is related to the angle between the sidewall and the incident flux, the deposition rate changes as the NW bends and also varies along the length of a bent NW. The predicted shell thickness and local bending radius along the NW were calculated using an iterative approach, approximating the NW core to have a circular cross section. In this model, the 40 nm thickness (planar deposition) was divided into 100 deposition steps and the NW was divided into segments of 25 nm length, each having a constant deposition rate. For each deposition step, the local deposition was calculated (taking bending into account) and the curvature of the segment was calculated using the analytic model of Lewis *et al.* (2018 ▸). For sample 1, since the lattice mismatch for the 5 nm GaAs and 10 nm GaAs/Al_0.3_Ga_0.7_As components is negligible and the InAs sub-shell of 2 nm is very thin, we combine all these shells and assume a GaAs core of 111 nm thickness for the simulations. The calculations predict that the shell thickness increases significantly from 27 nm at the base to 45 nm near the top of the bent NW, and the bending radius varies from 3080 nm at the base to 2980 nm near the top. For sample 2, the shell thickness also varies from 27 nm at the base to 45 nm near the top, and the local bending radius varies from 1990 nm at the base to 2080 nm near the top. We note that the predicted radius for both samples is significantly smaller than what is observed experimentally. This could be either due to an overestimation of the shell thickness or because the shell growth is considered to be pseudomorphic. Plastic relaxation is, however, present at the core–shell interface as we observed the diffraction signal of the shell with a different lattice parameter in Fig. 3 ▸. Nevertheless, the model confirms that an inhomogeneous shell thickness causes a slight variation of the bending radius leading to higher bending near the top, qualitatively in agreement with our experimental observations. We note that reducing the asymmetric shell thickness in the model increases both the average bending radius and the variation in radius along the NW.

In order to assess the strain state of the NW core we look at the comparison of the experimental data with X-ray diffraction simulations. We showed in the theory section that the width of the diffraction signal for purely elastic bending and our experimental parameters can be associated with the bending. For the simulation curves shown in Fig. 11 ▸ we used the nominal NW thickness, which was found to agree well with the thickness fringes observed in our RSMs. Again we approximate the entire core and symmetric shell structure of sample 1 as a GaAs core. Having fixed the NW geometry the only relevant parameter which remains is the NW bending radius. For the simulation curves for sample 1 [Figs. 11 ▸(*a*) and 11 ▸(*b*)] we find that a radius of 11.0 ± 0.5 µm explains the observed width of the diffraction curves well. The radius is in good agreement with that determined earlier and that seen in the scanning electron microscope images. This suggests that the deformation of the NW core is indeed fully elastic without signs of plastic deformation inside the core.

In order to find an agreement for the peak position we had to shift the diffraction curve by approximately 0.005 Å^−1^ towards smaller *q* values. The reason for this could be twofold. Either a small amount of WZ phase mixed into the NW or the asymmetric placement of the shell with larger lattice parameter can explain this. Since the partially grown shell causes tensile strain of the NW on the side it is attached to, which has no counterpart on the opposing side, the overall strain in the NW is more tensile. This means that the average lattice parameter in the NW is slightly larger than that of bulk GaAs used in the simulation. If all the shift of the diffraction curve in Fig. 11 ▸ corresponds to a change of the average lattice parameter it would amount to 0.014 Å. For the bimetallic strip scenario mentioned in the *Introduction* [Sec sec1] this scenario is consistent with the neutral line, *i.e.* the unstrained part of the core material, being located towards the far side of the partial shell. Overall this causes the peak of the NW to move slightly towards lower *q* values. Since in our simulations the neutral line is located in the center of the NW we have to mimic this offset by shifting the diffraction curve. Because we know from TEM investigations that some WZ phase might be present, it is likely that a combination of the two effects (WZ inclusion and asymmetric strain) determines the resulting shift of the diffraction peak.

For NW3 of sample 2 a bending radius of 3 µm leads to rough agreement between the calculated line profile and the experimental observations averaged over the full measured tilt range [Fig. 11 ▸(*c*)]. It can, however, be clearly seen that the experimental curves for tilt ranges corresponding to segments of the NW closer to the substrate interface are significantly narrower and therefore less bent, corresponding to a bending radius almost 1 µm larger. This suggests that the different bending radii seen in different parts of the TEM images indeed reflect an intrinsic variation of the bending radius within the NWs. The growth modeling, however, predicts only a much smaller variation of the bending radius due to the inhomogeneous shell thickness. Therefore, we speculate that in this sample not only the shell thickness but also the degree of plastic relaxation might change along the NW, potentially leading to a stronger change of the bending.

The bending radius determined from the peak width can also be converted to a change of strain from the facet in contact with the partial shell to the opposite side. For purely elastic bending this difference in strain is trivially given by 2ρ/*R*, which is ∼0.9% in NW1 and NW2 and ∼2.5% in NW3. Such high uniaxial strain values can otherwise only be produced by the method of strain redistribution in micro-bridges which are lithographically produced out of thin films.

## 7. Conclusion   

We have demonstrated measurement strategies and analysis of X-ray diffraction data for highly bent NWs in their as-grown geometry with micro-focus X-ray diffraction. By extending the kinematical X-ray scattering theory for circularly bent crystal structures, we performed model calculations and reached good agreement with experimental data. By comparing simulations with our experimental data we obtain the bending radii of individual NWs. Our results further provide insights into the homogeneity of the bending of the NWs along their growth axis and allow us to directly access the amount of strain in the NW core material. We have shown that the bottom part of the NWs can have significantly lower bending/strain. Model calculations suggest that this can be related to an inhomogeneous shell thickness along the NW. The amount of uniaxial strain present in the NWs is comparable to the highest strains reported in micro-brigdes but is present directly in the as-grown state.

## Supplementary Material

Supporting figures and text. DOI: 10.1107/S1600576720011516/to5214sup1.pdf
