# Supplementary material for: X-ray diffraction reveals the amount of strain and homogeneity of extremely bent single nanowires
Source: J Appl Crystallogr. 2020 Sep 23;53(Pt 5):1310–20. doi: 10.1107/S1600576720011516 (PMC7534542; doi:10.1107/S1600576720011516)
Supplement: Supplementary file 1 [file j-53-01310-sup1.pdf]

X-ray diffraction reveals the amount of strain and homogeneity of extremely bent single nanowires
—
Supplementary Material

Arman Davtyan,[a]*† Dominik Kriegner,[b]*† Václav Holý,[c] Ali AlHassan,[a] Ryan B. Lewis,[d,e] Spencer McDermott,[d] Lutz Geelhaar,[e] Danial Bahrami,[a] Taseer Anjum,[a] Zhe Ren,[f] Carsten Richter,[g]‡ Dmitri Novikov,[h] Julian Müller,[a] Benjamin Butz[a] and Ullrich Pietsch[a]

[a]Faculty of Science and Engineering, University of Siegen, D-57068 Siegen, Germany, [b]Institut für Festkörper- und Materialphysik, Technical University Dresden and Würzburg-Dresden Cluster of Excellence ct.qmat, Germany, [c]Department of Condensed Matter Physics, Charles University in Prague, Ke Karlovu 5, 121 16 Praha Czech Republic, [d]Department of Engineering Physics, McMaster University, L8S 4L7 Hamilton, Canada, [e]Paul-Drude-Institut für Festkörperelektronik, Leibniz-Institut im Forschungsverbund Berlin e.V., Berlin, Germany, [f]Synchrotron Radiation Research, Lund University, 221 00, Lund, Sweden, [g]ESRF – The European Synchrotron, 71 Avenue des Martyrs, 38000 Grenoble, France, and [h]Deutsches Elektronen-Synchrotron, PETRA III, Hamburg D-22607, Germany

Prior to NW measurements the X-ray wavefront was characterized by 2D Ptychography at forward direction. To this aim the available setup at ID01 using Siemens star and PyNX software for phase retrieval reconstructions was used. The detector to Siemens star distance was set to 1547 mm in order to capture the fine speckles originating from the interaction of coherent X-rays within the structure of Siemens star. For successful and better convergence as usual (from our experience) the Siemens star was mounted not at the focus but downstream by around 86 µm. Figure 3(a) represents the reconstruction of the Siemens star, amplitude and phase image, and indicates the trust region by black cycle. Figure 3(b) represents the reconstructed wavefront at the position of the Siemens star, and figure s1(c) at the focus. As it can be seen the Intensity modulations present in the profile of the incoming X-ray beam focused by KB mirrors at 9KeV. The line profiles of the wavefront are presented in figure s1(d) where we observe the beam with FWHM of 480x230 nm^2 (HxV). The phase retrieval algorithm uses the sequence of algorithms and probe activation following to: 20DM, probe=1, 100DM, 100AP, 100ML, nprobe=3, 100DM, 100AP, 100ML using 393 frames and with the pixel size (resolution) of 14.7 *nm*.

† contributed equally
‡ current address: Leibniz-Institut für Kristallzüchtung, Berlin, Germany

supplementary material

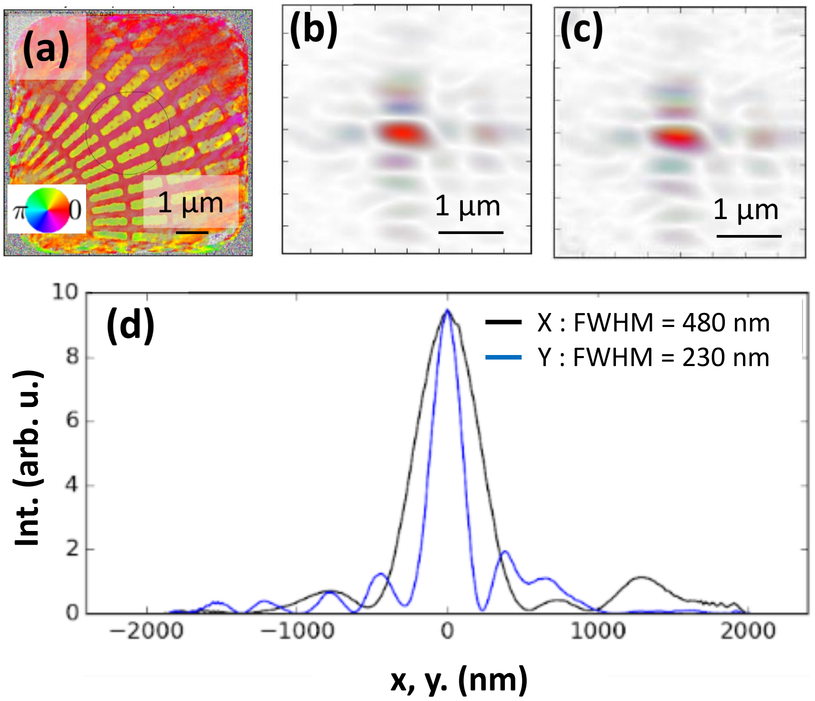

Figure S1
Beam profile/wavefront characterization from ID01 beamline. (a) Reconstructed amplitude and phase image of Siemens star. (b,c) Reconstructed amplitude and phase image of beam profile out of focus and in focus positions. (d) Line profiles of the X-ray beam at focus along the horizontal and vertical directions.

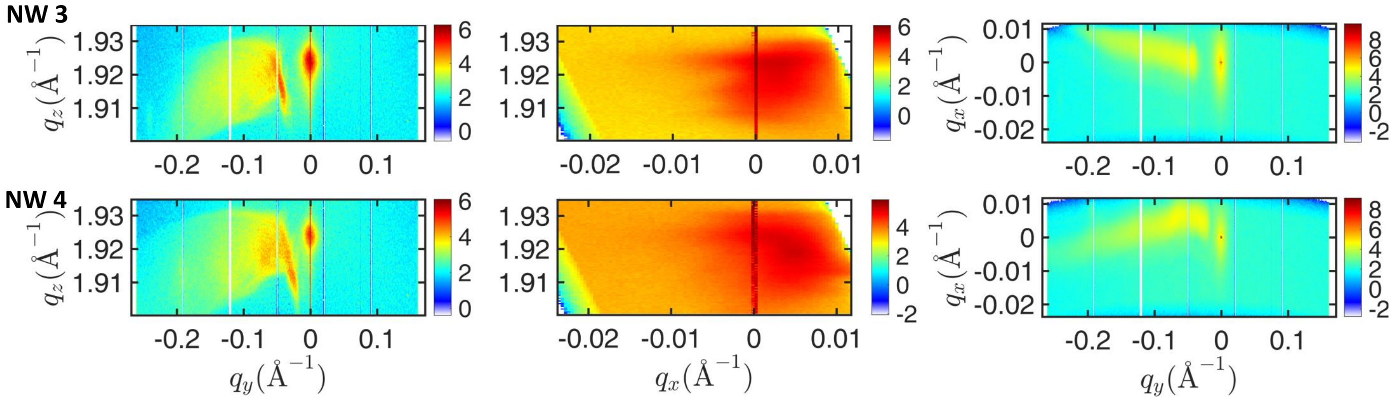

Figure S2
Projections of the 3D RSMs measured from the basis of NW3 and NW4 of sample 2. The NW signal left of the substrates CTR starts from $q_y \sim -0.03$ Å^{-1} and extends to lower q_y values. The offset of the NW signal from the CTR even for the bottom part of the NW means that these NWs are already nucleating with a slight tilt with respect to the substrate (111) surface.

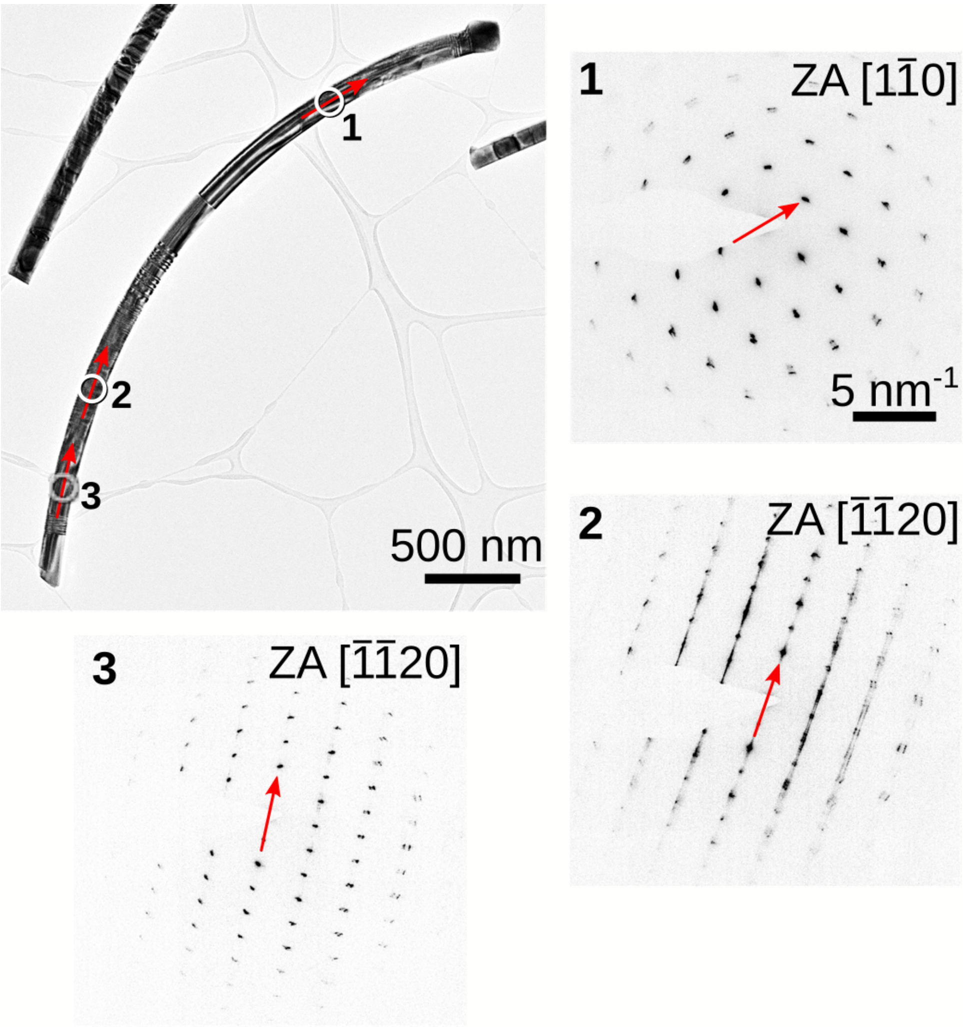

Figure S3
Three transmission electron diffraction patterns recorded from a NW of sample 2. The location from which the diffraction patterns were recorded are marked in the overview image shown on the top right. The local wire axis is found to correspond with the $[111]_{ZB}$ or $[0001]_{WZ}$ in the wire axis.
